# Not Only Redox: The Multifaceted Activity of Cerium Oxide Nanoparticles in Cancer Prevention and Therapy

**DOI:** 10.3389/fonc.2018.00309

**Published:** 2018-08-14

**Authors:** Francesca Corsi, Fanny Caputo, Enrico Traversa, Lina Ghibelli

**Affiliations:** ^1^Department of Chemical Science and Technologies, University of Rome Tor Vergata, Rome, Italy; ^2^Department of Biology, University of Rome Tor Vergata, Rome, Italy; ^3^School of Materials and Energy, University of Electronic Science and Technology of China, Sichuan, China

**Keywords:** cerium oxide nanoparticles, redox-independent, cancer treatment, cancer prevention, antioxidant, radio-protection, radio-sensitization, tumor microenvironment

## Abstract

Much information is accumulating on the effect of cerium oxide nanoparticles (CNPs) as cell-protective agents, reducing oxidative stress through their unique ability of scavenging noxious reactive oxygen species *via* an energy-free, auto-regenerative redox cycle, where superoxides and peroxides are sequentially reduced exploiting the double valence (Ce^3+^/Ce^4+^) on nanoparticle surface. *In vitro* and *in vivo* studies consistently report that CNPs are responsible for attenuating and preventing almost any oxidative damage and pathology. Particularly, CNPs were found to exert strong anticancer activities, helping correcting the aberrant homeostasis of cancer microenvironment, normalizing stroma-epithelial communication, contrasting angiogenesis, and strengthening the immune response, leading to reduction of tumor mass *in vivo*. Since these homeostatic alterations are of an oxidative nature, their relief is generally attributed to CNPs redox activity. Other studies however reported that CNPs exert selective cytotoxic activity against cancer cells and sensitize cancer cells to chemotherapy- and radiotherapy-induced apoptosis: such effects are hardly the result of antioxidant activity, suggesting that CNPs exert such important anticancer effects through additional, non-redox mechanisms. Indeed, using Sm-doped CNPs devoid of redox activity, we could recently demonstrate that the radio-sensitizing effect of CNPs on human keratinocytes is independent from the redox switch. Mechanisms involving particle dissolution with release of toxic Ce^4+^ atoms, or differential inhibition of the catalase vs. SOD-mimetic activity with accumulation of H_2_O_2_ have been proposed, explaining such intriguing findings only partially. Much effort is urgently required to address the unconventional mechanisms of the non-redox bioactivity of CNPs, which may provide unexpected medicinal tools against cancer.

## Introduction

Materials acquire peculiar activities at the nanoscale (1–100 nm), due to their increased reactive surface/bulk ratio with respect to larger structures: for example, gold, essentially inert in the bulk, becomes highly reactive in the form of nanoparticles, displaying catalytic activity (1). Industrial exploitation of nanomaterials allows unprecedented applications in almost every field, including important biomedical applications. In addition to the well-recognized use of tailored nanostructures for drug delivery (2), nanomedicine can indeed exploit intrinsically bioactive nanoparticles as effective medicinal tools where, rather than being an inert platform, the material itself acts as the therapeutic agent (3, 4). In particular, in clinical cancer research, bioactive materials are emerging as a possible tool to overcome the intrinsic limitations of conventional anticancer therapies.

Cerium oxide nanoparticles (CNPs) are receiving much attention for their unusual antioxidant properties, promising to act as potent antioxidant and anticancer drugs. Cerium is a rare earth element belonging to the lanthanide series, possessing a stable cerium (IV) oxidation state that coexists with cerium (III). In the nanoparticle form, cerium oxide atoms form a cubic crystalline fluorite lattice structure where Ce^3+^, and the compensating oxygen vacancies, localize at the nanoparticle surface (5). The double valence generates a redox couple responsible for a robust catalytic activity, widely exploited in industrial applications, including catalysis (6), UV screens (7), gas sensors (8), solar, and fuel cells (9, 10).

The medicinal appeal of CNPs is mainly due to their unprecedented auto-regenerative antioxidant activity, which can scavenge noxious reactive oxygen/nitrogen species (ROS/RNS) generated by exogenous or endogenous sources (11) by combining (i), a superoxide dismutase (SOD) mimetic activity, responsible for reducing superoxide or peroxynitrite to peroxide and nitrate (respectively) undergoing oxidation from Ce^3+^ to Ce^4+^ (12, 13), with (ii), a catalase mimetic activity, where Ce^4+^ is reduced back to Ce^3+^ by oxidizing hydrogen peroxide to molecular oxygen and water (14). Thus, CNPs undergo a complete, energy-free redox cycle, eliminating the most toxic ROS while regenerating the original redox status (15).

Here, we will review literature data reporting the cancer preventive and therapeutic potentials of CNPs. Intriguingly, they do not deal exclusively with antioxidant actions: non-redox activity of CNPs are indeed emerging, with mechanisms that still need to be understood, and that may provide CNPs with the potential to act as unconventional anticancer agents via multiple, unrelated mechanisms.

## Cancer preventing activity of CNPs

Cancer origin is mainly attributed to accumulation of mutation events, due to environmental mutagens including pollution and radiation, and endogenous disequilibria such as chronic inflammation. A main mediator of both is oxidative stress, thereby antioxidants, such as e.g., dietary vitamins, are precious sources of cancer preventing agents.

ROS-promoted damage is a major cause of cell and genetic alterations, and the basis of almost any pathology, including cancer (16); accordingly, much effort has been posed to identify antioxidant agents able to protect against oxidative stress and the related pathologies. However, no satisfactory antioxidant has been identified so far: the canonical molecular antioxidant proved being short-lasting and indiscriminate, eliminating also ROS acting as signaling molecules in many cellular pathways, thereby endangering the correct redox homeostasis and cell functioning.

In this scenario, CNPs act as long-lasting regulators of redox metabolism rather than simple scavengers, efficiently eliminating ROS only when required, thus preserving basal cell activities, proposing them as bio-compatible antioxidant tools (17).

In particular, CNPs were shown to exert a potent antioxidant action, preventing oxidative stress, cell damage and death by apoptosis (3, 15, 18). CNPs also affect the cellular consequences of oxidative imbalance, modulating the activity of redox-responding proteins, for example, inactivating the transcription factor NFκB (19) and the downstream signaling cascade, which are implicated in cancer genesis and progression. Thus, by scavenging ROS, CNPs may modulate many cellular signal transduction processes regulating stress response, cellular metabolism, proliferation and cell cycle checkpoints (20), and control homeostatic pathways, including those involved in cancer and other oxidative-related pathologies (21–26).

Intriguingly, other CNPs-dependent effects are reported, hardly compatible with antioxidant action. For instance, (27) reported in gastrointestinal epithelial cells the upregulation of SOD2 gene expression while exerting a ROS scavenging action, which is a paradoxical effect since antioxidant enzymes are generally downregulated by the presence of antioxidant agents. Again, (28) showed that CNPs can hydrolyze phosphate ester bonds in abiotic systems, potentially interacting with ATP and phosphorylated proteins also inside cells. Other effects involve particle dissolution in acidic environment with release of bioactive Ce^4+^ ions (29), or to differential sensitivity of the SOD- *vs-* catalase-mimetic activity to low pH (30), mainly dealing with induced toxicity, which can be exploited against cancer cells, as we will discuss later.

Radiotherapy and all diagnostic procedures involving X-rays pose serious risks for exposed individuals, causing direct and ROS-mediated toxicity; therefore, radio-protective agents, including antioxidants, ameliorate radiation-induced acute and delayed damage (4, 31). Preventing radiotherapy-induced death of healthy (i.e., non-cancer) cells is an important task; however, to avoid a paradoxical pro-mutagenic effect, efficient radio-protectors should not merely inhibit apoptosis, but also reduce genetic radiation damage. Importantly, CNPs strongly reduce UV-induced apoptosis and at the same time, they decrease DNA damage, accelerate repair, and abate mutagenesis (18), thus promising to be efficient and safe UV-protectors.

The antioxidant properties of CNPs have attracted attention as possible effective countermeasure against ionizing radiations. CNPs ability to protect tissues from radiation-induced damage was reported in many systems, for instance gastrointestinal epithelium, where CNPs act as direct ROS scavengers (27) or breast epithelial cells, where CNPs rescue almost 99% of normal irradiated cells: interestingly, no protection was provided to tumor cells (32). The reason for this very important selectivity was not investigated; conceivably, it may be linked to the differential toxicity toward cancer vs. normal cells, discussed below.

## CNPs as anticancer therapeutic agents

### Relief of tumor microenvironment malignant features

Tissues are composed not only of epithelial (i.e., tissue specific) cells, but also of “accessory” components such as blood vessel (endothelial cells), stroma fibroblasts, extracellular matrix, and a range of active biomolecules produced by all cell types, creating a complex signal network responsible for tissue functioning and homeostasis. Cancer genesis and progression is caused by homeostatic errors occurring within the tumor microenvironment (33), related or not with genetic mutations, dealing with all components of the cancer tissue (34), and implying many alterations, including increased oxidative status. ROS play a major role in promoting the aberrant cancer homeostasis, favoring vicious communications between cancer cells and stroma, endothelium, and matrix, thus favoring tumor neo-angiogenesis, matrix degradation, and improper immune infiltrations (35). Hence, antioxidant therapy is considered as a mean to prevent and revert the alteration of tumor microenvironment: CNPs have raised much attention in this regard.

As a matter of fact, CNPs administration at the tumor site helps correcting cancer microenvironment homeostasis in animal models (36), effect attributed to restoration of a proper redox asset. In fact, CNPs act more efficiently than canonical antioxidants: for example, SOD (or catalase, or the combination of the two), whose activity is mimicked by CNPs, is not as effective, protecting in the initial steps of carcinogenesis but promoting progression in advanced stages (37, 38). This suggests that either CNPs antioxidant action is “better” than the enzymatic one, or that they may exert additional, non-redox effects. For instance, (39) showed that CNPs inhibited the migration and proliferation of gastric cancer cells by transactivating the box helicase 15 (DHX15) and its downstream MAPK signal pathway without affecting ROS levels. Therefore, when the role of CNPs in tumor microenvironment are mechanistically investigated, non-redox effects begin to emerge.

Stroma changes occurring during tumorigenesis include the trans-differentiation of fibroblasts into myofibroblasts, modulated by cytokines such as tumor transforming factor beta 1 (TGFβ1) released by tumor cells, implying an oxidative cascade (34). CNPs efficiently inhibit myofibroblast formation and localization at the tumor front, preventing promotion of tumor growth, and invasion (40); the effects were attributed to CNPs redox switch. However, also in this case, CNPs were shown to contrast myofibroblast formation without altering ROS level (41).

Promotion of endothelial cell proliferation, generating new blood vessels for feeding and sustaining tumor growth and invasion (35), occurs through redox-sensitive angiogenic growth factors, including vascular endothelial growth factors (VEGF), fibroblast growth factor (FGF), and their receptors (42). CNPs were shown to efficiently contrast angiogenesis in ovarian carcinoma mouse model (43), attenuating VEGF-mediated proliferation of human umbilical vein endothelial cells, and inhibiting VEGF-induced matrix metalloproteinase 2 activity, clearly inhibiting VEGF mediated downstream signaling.

These effects on tumor microenvironment seem not only circumstantial, rather possibly leading to the real control of tumor growth: indeed, many studies report that administration of CNPs in tumor-bearing mice causes tumor reduction (27, 36), which is a logical consequence of restoration of a more correct microenvironment.

### Direct and selective killing of cancer cells

In light of CNPs antioxidant activity, usually resulting in protective effects against oxidant-promoted apoptosis (15, 18, 44), it is difficult to consider CNPs as cytotoxic agents able to kill cancer cells, as often proposed. Nevertheless, in some instances CNPs do act as pro-oxidant and pro-apoptotic agents. For instance, (36) reported that CNPs induced apoptosis on melanoma, but not on stroma cells; this is related to selective ROS production, leading to mitochondria dysfunction (45). The finding that CNPs exerted similar pro-apoptotic effects on other cancer cells (46), led to hypothesize a differential effect on normal vs. cancer cells, which was attributed to the increased acidification of cancer microenvironment, which would turn CNPs into toxic agents. It is known that at pH ≤ 4 the catalase, but not the SOD-mimetic activity of CNPs is inhibited (30), with consequent accumulation of H_2_O_2_, more toxic than superoxides: in these conditions, CNPs would act as pro-oxidants. Moreover, CNPs release toxic Ce^4+^ ions due to nanoparticle acidic dissolution at pH ≤ 4. Attributing CNPs anticancer effect to strong pH decrease is however nonsense: the tumor microenvironment is indeed more acidic than normal tissues due to the Warburg effect (47, 48), but only of a few decimals, and cannot reach pH 4: this would lead to immediate cell and tissue collapse. However, pH 4 is reached within lysosomes, intracellular organelles that increase in volume and activity in cancer cells (49). This may favor CNPs lysosomal localization (50), implying that CNPs dissolution and/or H_2_O_2_ accumulation may occur to a greater extent in cancer than in normal cells. However, (51) did not find any correlation between CNPs lysosomal localization and intracellular ROS modulation in human ovarian and colon cancer cells.

A literature survey reveals so many exceptions to the pseudo-rule of CNP-selective killing of cancer cells, with examples of cancer cells protection against induced apoptosis [e.g., (15, 52, 53)], and of normal cells killing [e.g., (54)], to question the universality of the selective cytotoxicity. Rather, the pro- or anti-apoptotic effect of CNPs may depend on individual cell sensitivities, independently of being normal or cancerous, possibly consisting of different lysosomal trafficking, favoring or not Ce^4+^ release or H_2_O_2_ accumulation. The cancer cell selective killing may be then an epiphenomenon, suggesting that CNPs anticancer effects rather rely on microenvironment control.

### Radio-sensitization

Beside surgery, radiotherapy with ionizing radiation remains the standard care for many advanced carcinomas, either alone or in combination with other therapies; the rationale is promoting cell killing by apoptosis *via* direct radiation damage (i.e., promotion of double-strand DNA breaks) or through radiation-induced ROS (single-strand DNA breaks and protein and lipid peroxidation). Unfortunately, many tumor cells become radio-resistant as part of tumor progression, therefore it is necessary to use adjuvant treatments favoring radiation-induced cell death (55, 56). CNPs are potential radio-sensitizing agents acting through different strategies.

One strategy consists in enhancing radiation toxic effect at the tumor site: nanoparticles made of high atomic number materials, including CNPs, when irradiated with specific energy beams, emit ROS or heat, causing a “dose-enhancement effect” (4, 57, 58) leading to extra toxicity on cells present in the treated area. An additional effect was proposed for CNPs, where X-rays induce a pH-mediated dissolution in aqueous media, resulting in the release of the toxic Ce^4+^ ions (29).

Wason et al. (59) proposed a CNPs-dependent selective, acidic-mediated increase of radio-toxicity against cancer cells; however, it remains hard to hypothesize that pH levels ≤4 may be reached even in irradiated contexts.

CNPs-induced sensitization includes other cytotoxic treatments, *e.g.*, they enhance toxicity of doxorubicin, a DNA-damaging chemotherapeutic drug, on melanoma cells via ROS production (60).

**Figure 1:**
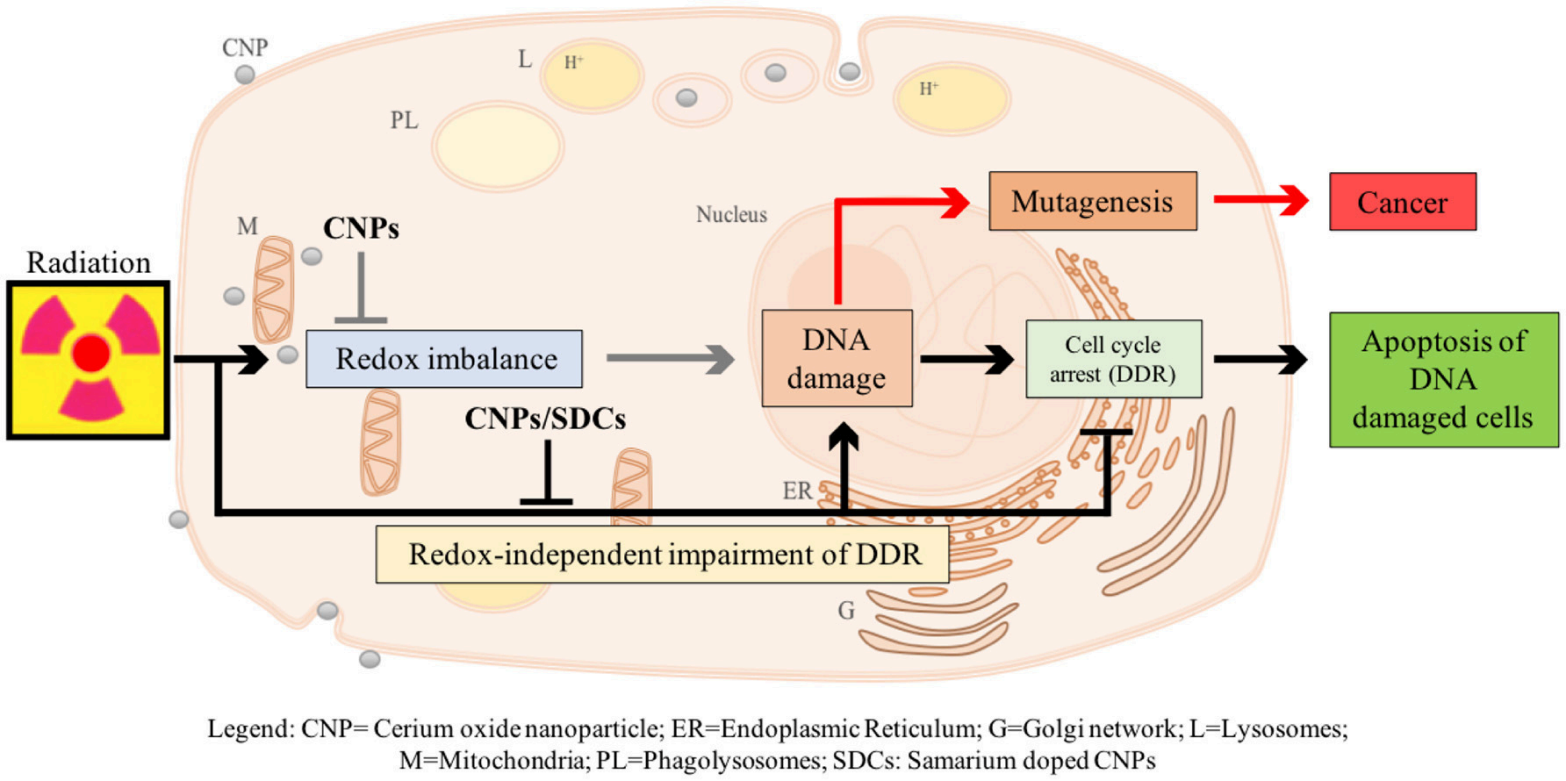
Proposed model of action of CNPs as redox-independent radio-sensitizing agents in HaCat keratinocytes cells. CNPs administration may promote in a redox-independent fashion the strengthening of cell DNA damage response (DDR) after exposure to radiations, diminishing X-ray-induced DNA lesions on one side, and increasing the stringency of cell cycle checkpoints and forcing damaged cells to undergo apoptosis on the other, thus preventing radiation-induced mutagenesis.

We have recently described a radio-sensitization effect of CNPs, showing that they increase X-ray-induced apoptosis on HaCat keratinocytes, without affecting untreated cells (Caputo et al., submitted)^1^. However, this is not due to H_2_O_2_ accumulation: on the contrary, CNPs restore catalase activity destroyed by X-rays, preventing radiation-promoted ROS and DNA damage. Searching for a mechanism, we observed that CNPs, in spite of reducing DNA breaks, improved the efficiency of the cell perception of/reaction to, DNA damage, increasing the apoptotic outcome *via* damage-unrelated mechanisms. In fact, CNPs restored DNA integrity checkpoints, generally lost in cancer cells, thereby almost abolishing X-ray-induced mutagenesis, by acting on the intimate pathways controlling survival of injured cancer cells. Intriguingly, this radio-sensitization is independent from CNPs redox switch because it was unaffected by Sm-doping, a strategy preventing the Ce^3+^/Ce^4+^ switch and the correlated antioxidant action providing stable 3+ valence (15, 18) (Figure 1).

Overall, the abiotic dose-enhancement effect and the biological regulatory role of restoring cell integrity checkpoints seem very promising strategies to exploit CNPs as radio-sensitizing devices.

## Conclusions

The survey we have presented here shows that combining CNPs with radiation or conventional chemotherapeutics may represent a novel anticancer strategy, helping re-modulating cancer microenvironment, killing tumor cells while sparing normal ones, thus improving the therapeutic outcome. So far, such appealing potentialities are limited to research aspects: is it conceivable that CNPs may turn into real therapeutic tools?

CNPs toxicity issues were abundantly investigated (3); overall, CNPs are considered biocompatible agents, rapidly cleared from organs (61) with very little toxicity (62, 63); in fact, CNPs play a substantial role as protectors against induced damage (64). However, the implications of nanoparticle-organism interactions in therapeutic perspectives are still a highly debated issue, generally considered a hazard, even though, pharmacologically, cell internalization of bioactive nanoparticles may provide an extra bonus, allowing persistence of the therapeutic effect for long time after the initial administration (65), avoiding the necessity of chronic treatments as required for molecular drugs, exerting only transient effects. Nevertheless, it is hard to foresee a rapid approval of CNPs clinical usage, apart perhaps for topic applications, apparently devoid of risk.

**Figure 2:**
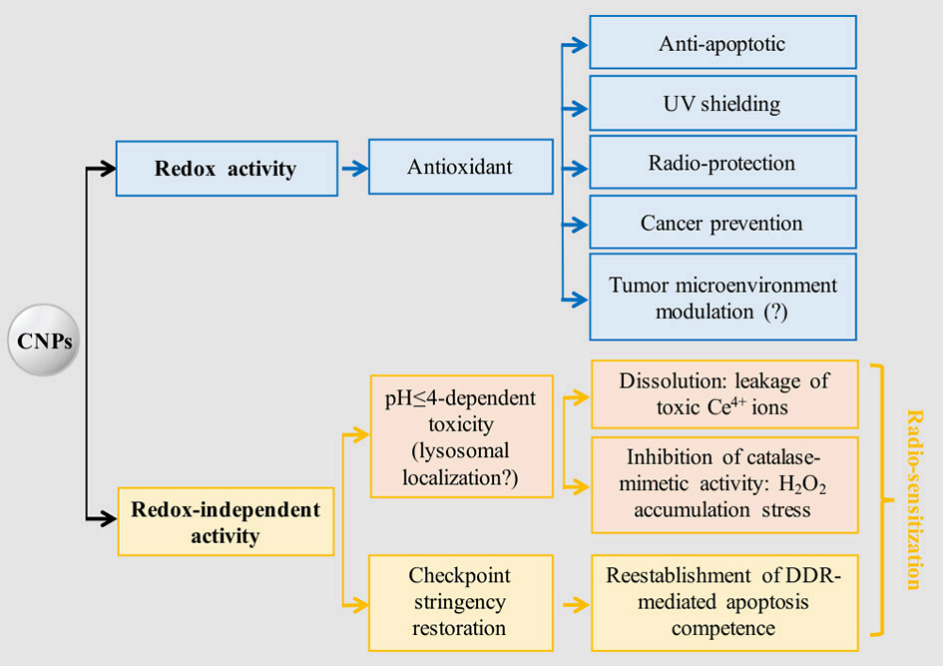
CNPs main redox-dependent and independent biological effects. Tumor microenvironment modulation supposedly occurs via antioxidant effect, but no experimental evidence is available. It is hypothesized that acidic-induced toxicity occurs in lysosomes, being the only biological site where a suitable pH (≤4) is reached; however, no experimental evidence is available. Radio-sensitization may be achieved through toxic and non-toxic activation of the apoptotic program, the latter being selective for cancer cells possessing defective DNA integrity checkpoints. DDR, DNA damage response.

CNP abilities to act against different cancer features are diverse and occur through disparate mechanisms, making CNPs multifaceted, pleiotropic, and non-conventional anticancer tools. A very intriguing aspect is the multiple, un-related non-redox effects, spanning from leakage of toxic ions, to the paradoxical oxidative stress due to the differential inhibition of catalase- vs- SOD-mimetic activity, to the still ill-defined ability to restore cell-integrity checkpoints. In comparison, the antioxidant activity implying the self-regenerating redox state appears straightforward, explaining, in a univocal sense, antioxidant, anti-apoptotic, and environmental protective effects (Figure 2).

The fact that CNPs may affect cell survival in two opposite ways, reducing the extent of damage-induced apoptosis on one side, and promoting apoptosis restoring cell integrity checkpoints on the other, may at first appear paradoxical. However, it must be considered that apoptosis is not only the result of induced damage, but also a physiological response to supernumerary or dangerous cells, induced to die by purely signaling activities, e.g., *via* activation of the Fas/Fas ligand system (66), or p53 activation (67): apoptosis is thus a way to free the organism of possibly mutated cells. In this scenario, the two effects are not one the reverse of the other, but the result of two different, unrelated anticancer actions of CNPs. Cancer cells, as a rule, lose cell integrity check-points during tumor progression, thereby surviving and replicating in spite of DNA damage (68), increasing malignancy. Agents that restore the signaling responsible for checkpoints induction, can re-establish apoptosis competence without direct toxicity: therefore, CNPs are radio-sensitizers in the strictest sense. It will be important to uncover the mechanism of this intriguing effect and explore whether it can be at the basis also of the other examples of CNPs-dependent radio-sensitization reported.

Finally, it must be underscored that even if the effects mediated by CNPs (e.g., on cancer microenvironment) are apparently redox-mediated, this must be proven experimentally: cell signaling pathways are so much intersecting, that it is risky to attribute mechanisms *a priori*. The tool of inhibiting CNPs antioxidant activity by Sm doping is a straightforward way to simply address this point, and may help in unequivocally describing the mechanisms at the basis of the diverse anticancer activities of CNPs.

## Author contributions

The elaboration of the concepts reported in this Perspective paper comes from the joint effort of FrC, FaC, ET, and LG. FrC and LG wrote the paper, which was discussed and approved by all authors.

### Conflict of interest statement

The authors declare that the research was conducted in the absence of any commercial or financial relationships that could be construed as a potential conflict of interest.

## Footnotes

^1^Caputo F, Giovannetti A, Corsi F, Maresca V, Briganti S, Licoccia S, et al. Cerium oxide nanoparticles re-establish cell integrity checkpoints and apoptosis competence in irradiated HaCat cells via novel redox-independent activity. *Front Pharmacol*. (2018).
